# Modeling and analysis of the Delta-Notch dependent boundary formation in the *Drosophila* large intestine

**DOI:** 10.1186/s12918-017-0455-8

**Published:** 2017-09-21

**Authors:** Fei Liu, Deshun Sun, Ryutaro Murakami, Hiroshi Matsuno

**Affiliations:** 1 Control and Simulation Center, Harbin Institute of Technology, West Dazhi Street 92, Harbin, 150001 People’s Republic of China; 2 School of Software Engineering, South China University of Technology, Building B7, Guangzhou, 510006 People’s Republic of China; 3 Faculty of Science, Yamaguchi University, Yoshida 1677-1, Yamaguchi, 753-8512 Japan

**Keywords:** Boundary formation, *Drosophila* large intestine, Delta-Notch signaling pathway, Local stability analysis, Simulation validation, Perturbation analysis

## Abstract

**Background:**

The boundary formation in the *Drosophila* large intestine is widely studied as an important biological problem. It has been shown that the Delta-Notch signaling pathway plays an essential role in the formation of boundary cells.

**Results:**

In this paper, we propose a mathematical model for the Delta-Notch dependent boundary formation in the *Drosophila* large intestine in order to better interpret related experimental findings of this biological phenomenon. To achieve this, we not only perform stability analysis on the model from a theoretical point of view, but also perform numerical simulations to analyze the model with and without noises, the phenotype change with the change of Delta or Notch expression, and the perturbation influences of binding and inhibition parameters on the boundary formation.

**Conclusions:**

By doing all these work, we can assure that our model can better interpret the biological findings related to the boundary formation in the *Drosophila* large intestine.

## Background

The large intestine of *Drosophila* embryo is a middle and large region of the *Drosophila* hindgut and is subdivided into dorsal and ventral domains, between which a one-cell-wide strand of boundary cells forms bilaterally for wild-type embryos [1–4]. The large intestine is a multicellular system that involves a number of cells composed of three cell types, dorsal, ventral and boundary cells, organized in a single-layered epithelial tube [5]. For such developmental patterning problems, different kinds of computational strategies [6] have been proposed, e.g., signaling gradients [7] and activator-inhibitor systems [8], and many computational techniques are adopted, e.g., ordinary/partial differential equation [9] and colored Petri nets [10]. See [11] for a review. However, for the boundary formation in the *Drosophila* large intestine, the mechanism has been widely explored in vivo, e.g., [1–4, 12], but rarely from the computational point of view, e.g., [13].

It has been shown that the Delta-Notch signaling pathway plays an essential role in the boundary formation of the *Drosophila* large intestine [1, 12]. In fact, the Delta-Notch pathway is considered as one of the six major signaling pathways in cells, which is active in developing embryos at different phases [14]. Both Notch and Delta proteins are transmembrane proteins, where Notch proteins act as receptors and Delta proteins as ligands. When Delta ligands in a cell bind to Notch receptors in neighboring cells, all the cells in a system may evolve and finally form different types of patterns [15–18].

In order to understand the mechanism of the Delta-Notch pathway, there have been some mathematical models proposed. For example, Collier et al. proposed a simple ordinary differential equation (ODE) model for the Delta-Notch signaling pathway, and discussed numerical simulation of multiple-cell systems [19]. Boareto et al. devised a theoretical framework that includes a couple of ODEs to explore the effects of Jagged in cell-fate determination [20]. Sprinzak et al. gave a model of a set of ODEs for describing mutual inactivation of Notch and Delta and used this model to illustrate how cis-interactions between Notch and Delta generate mutually exclusive signaling states [21]. Specifically, Matsuno et al. analyzed the mechanism of the Notch-dependent boundary formation in the *Drosophila* large intestine and built a hybrid Petri net model to numerically explore how the Delta-Notch pathway affects the boundary formation in two-dimensional space [13].

In this paper, we propose a mathematical model for the Delta-Notch dependent boundary formation in the *Drosophila* large intestine based on the work of [13], aiming at better interpreting related biological findings and further making predictions. Compared with the existing work in this area, our work has the following main contributions.

(1) We give a mathematical model for the Delta-Notch dependent boundary formation in the *Drosophila* large intestine, which can better interpret relevant biological findings, so far obtained in the lab. Moreover, this model can be numerically simulated efficiently. To make the model both mathematically and biologically sound, we do the following analysis work.

(2) We perform local stability analysis on the two-cell model to make the model mathematically sound. The analysis confirms that the model would reach an equilibrium, which corresponds to that the system would result in a stable pattern in either normal or mutant conditions. The model is also stable when some conditions are satisfied, which means even if there are small disturbances of parameters (e.g., small environmental noises), the system would still converge to a stable state after a period of time. We use the local stability analysis result to determine appropriate parameter values that make the system stable.

(3) We perform numerical simulations with and without noises of Notch expression, which shows that both deterministic and random simulation results are consistent with experimental observations. We further analyze how phenotypes change with the change of Delta or Notch expression.

(4) We analyze the perturbation influences of binding and inhibition parameters on the boundary formation. As the boundary formation is affected by many environmental factors or noises, to make the model more realistic, we need to consider noises into the model by finding those parameters that have significant influences on the dynamics of the model when they vary. According to the analysis result, we add appropriate random noise items to key parameters of the model.

This paper is structured as follows. In the section of methods, we introduce relevant biological background of the Delta-Notch dependent boundary formation in the *Drosophila* large intestine, and present a mathematical model for this biological phenomenon. In the section of results and discussions, we give stability analysis, simulation analysis, and parameter perturbation analysis of the model. Finally, the conclusion is given.

## Methods

In this section, we introduce relevant biological background and give a description of the mathematical model we develop, as well as the simulation and analysis methods which we use.

### Boundary formation in the *Drosophila* large intestine

**Fig. 1:**
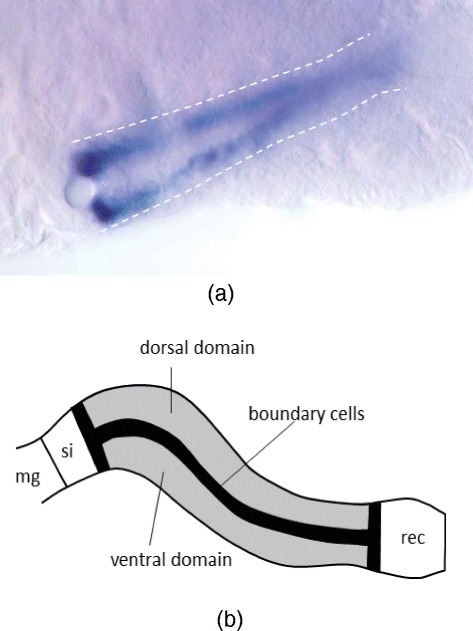
An illustration of the *Drosophila* hindgut. **a** Boundary cells form one-cell-wide domains bilaterally (*arrows*) between dorsal and ventral domains of the large intestine. **b** A diagram of the hindgut domains in the wild-type *Drosophila* embryo, adapted from [3]

The large intestine of *Drosophila* embryo is a major middle region of the *Drosophila* hindgut and is subdivided into dorsal and ventral domains (see Fig. 1), each of which is characterized by different cell types, dorsal or ventral. Between these two domains, a one-cell-wide strand of boundary cells forms in wild-type embryos.

**Fig. 2:**
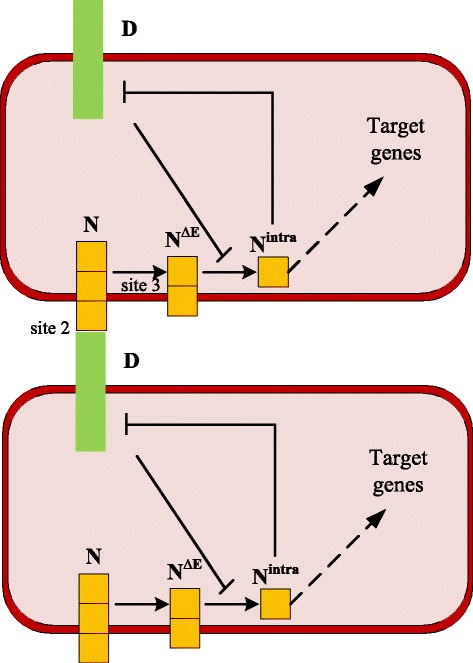
A diagram of the Delta-Notch signaling pathway in boundary cell formation of the large intestine. This pathway shows the interaction of two neighboring cells, which is adapted from [1]

The Delta-Notch signaling pathway plays an essential role in the formation of boundary cells [1, 12]. The main processes [1] are shown in Fig. 2. When the Delta ligands on the ventral cell surface bind to the Notch receptors of a neighboring Delta-negative dorsal cell, a Delta-Notch signaling cascade is activated. First the site 2 cleavage of the Notch proteins occurs to generate a transmembrane form (N^*Δ*E^), followed by the Presenilin-dependent site 3 cleavage, producing an active Notch intracellular fragment (N^intra^). N^intra^ then activates the target genes, inducing boundary cell differentiation. Moreover, Delta autonomously blocks the Presenilin-mediated site 3 cleavage and thus inhibits Notch signal transduction within Delta-positive ventral cells.

**Fig. 3:**
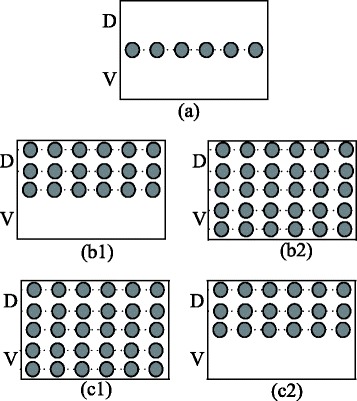
Diagram for the experimental result of the boundary formation of the *Drosophila* large intestine. (**a**): Wild-type, (**b1**) to (**b2**): over-expression of Notch with decreasing Delta background, and, (**c1**) to (**c2**): over-expression (decreasing) of Notch with fixed Delta background. Each filled circle is a boundary cell. *D* means the dorsal domain and *V* the ventral domain. This diagram is made according to [1, 4, 13]
- **Scenario 2.** In an embryo where over-expression of Notch or Delta proteins happens, the number of boundary cells would change. If we fix over-expression of Notch proteins, the number of boundary cells would increase with the decrease of Delta background. See Fig. 3 b1 to b2 for an illustration.
- **Scenario 3.** However, if we fix the background of Delta proteins, the number of boundary cells would increase with the increase of over-expression of Notch proteins. See Fig. 3 c1 to c2 for an illustration.
- **Scenario 4.** During the boundary formation in the *Drosophila* large intestine, environmental factors such as temperature may heavily affect the boundary formation, thus resulting in different (random) phenotypes. Therefore, the model to be built should incorporate such random noises into some parameters of interest.

In this paper, we aim to interpret the publicated findings about the boundary cell patterning in the large intestine [1, 4, 13] (see Fig. 3) and hypothesize the following scenarios for our model.

- **Scenario 1.** In a wild-type embryo, a one-cell-wide strand of boundary cells forms at the interface of dorsal and ventral domains. See Fig. 3a for an illustration.

In what follows, we will construct and analyze our model step by step.

### Mathematical model

**Fig. 4:**
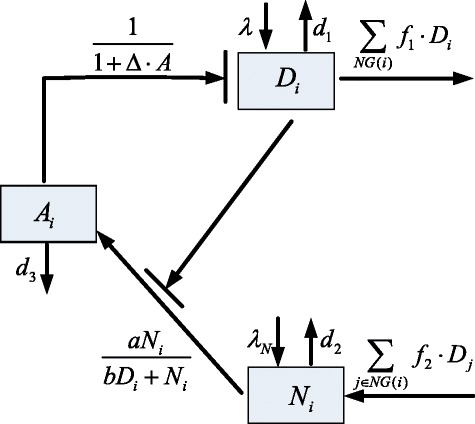
The model for one cell. In this model, only two species, Delta (*D*) and Notch are considered. Notch can be in inactive state (*N*) or active state (*A*)

Cell-to-cell interaction mediated by the Delta-Notch signaling pathway plays an essential role in the development of a multicellular organism such as the *Drosophila* large intestine. In this work we propose a mathematical model for the core part of the Delta-Notch dependent boundary formation in the *Drosophila* large intestine (see Fig. 4 for a one-cell model) based on the work of [13]. That is, we only consider two key species, Delta (*D*) and Notch, and Notch can be in the inactive state (*N*) or active state (*A*). Inactive Notch can be converted into the active state when coupled with Delta from neighboring cells. Active Notch can inhibit the production of Delta of the same cell through target genes. Besides, Delta also inhibits the production of Notch of the same cell.

**Fig. 5:**
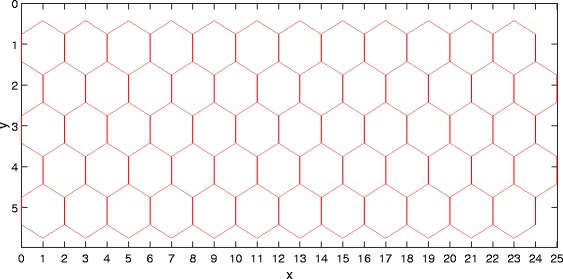
A two-dimensional lattice for the model. The mathematical model proposed in this paper works on the two-dimensional lattice, which defines a patch of a tissue

We arrange all cells in a regular *M*×*N* lattice (see Fig. 5 for an illustration), each site (cell) being a hexagon and having at most six neighboring cells. That is, we have *N* *C*=*M*×*N*/2 cells for an *M*×*N* lattice.

The mathematical model is then given as follows:

1 $$\begin{array}{@{}rcl@{}} \left\{ \begin{aligned} & \frac{d{{D}_{i}}}{dt}=\frac{\lambda }{1+\Delta \cdot {{A}_{i}}}-{{d}_{1}}{{D}_{i}}-\sum\limits_{NG(i)}{{{f}_{1}}\cdot {{D}_{i}}}, \\ & \frac{d{{N}_{i}}}{dt}={{\lambda }_{N}}-{{d}_{2}}{{N}_{i}}+\sum\limits_{j\in NG(i)}{{{f}_{2}}\cdot {{D}_{j}}}-\frac{a{{N}_{i}}}{b{{D}_{i}}+{{N}_{i}}}, \\ & \frac{d{{A}_{i}}}{dt}=-{{d}_{3}}{{A}_{i}}+\frac{a{{N}_{i}}}{b{{D}_{i}}+{{N}_{i}}}. \\ \end{aligned} \right. \end{array} $$

Here, 1≤*i*≤*N* *C*. *D*_*i*_, *N*_*i*_ and *A*_*i*_ denote the concentration of Delta proteins, inactive and active Notch proteins, respectively, in the *i* ^*th*^ cell. In $\frac {\lambda }{1+\Delta \cdot {{A}_{i}}}$, *λ* represents the production rate of Delta proteins, and *Δ* the inhibition coefficient of active Notch proteins *A*_*i*_. *d*_1_ represents the degradation rate of Delta proteins. $\sum \limits _{NG(i)}{{{D}_{i}}}$ represents the concentration of the Delta proteins (in the *i* ^*th*^ cell) that bind to the Notch proteins of the contacting cells of the *i* ^*th*^ cell, and *N* *G*(*i*) denotes all the neighbors of the *i* ^*th*^ cell. *f*_1_ represents the binding rate of Delta ligands to Notch receptors. *λ*_*N*_ and *d*_2_ represent the production rate and the degradation rate of inactive Notch proteins, respectively. *f*_2_ represents the binding rate of Delta ligands to Notch receptors. In $\frac {a{{N}_{i}}}{b{{D}_{i}}+{{N}_{i}}}$, *a* represents the conversion rate of Notch proteins from the inactive state to the active state, while *b* describes the inhibition effect of Delta on Notch in the same cell *i*. *d*_3_ is the degradation rate of active Notch proteins.

### Simulation and analysis methods

We encode our mathematical model and perform all simulations with Matlab 2016. Besides, we employ the following analysis methods for our model.

#### Local stability analysis

We conduct local stability analysis to explore stability conditions of our model, with which we further determine values of parameters.

A set of autonomous ordinary differential equations can be written in the following vector form:

$$\dot{x}=f(x) $$

where *x*=(*x*_1_,*x*_2_,…,*x*_*n*_) is the state vector and *f*=(*f*_1_,*f*_2_,…,*f*_*n*_). The Jacobian matrix is:

$$J=\left[ \begin{array}{cccc} \frac{\partial {{f}_{1}}}{\partial {{x}_{1}}} & \frac{\partial {{f}_{1}}}{\partial {{x}_{2}}} & \cdots & \frac{\partial {{f}_{1}}}{\partial {{x}_{n}}} \\ \frac{\partial {{f}_{2}}}{\partial {{x}_{1}}} & \frac{\partial {{f}_{2}}}{\partial {{x}_{2}}} & \cdots & \frac{\partial {{f}_{2}}}{\partial {{x}_{n}}} \\ \vdots & \vdots & {} & \vdots \\ \frac{\partial {{f}_{n}}}{\partial {{x}_{1}}} & \frac{\partial {{f}_{n}}}{\partial {{x}_{2}}} & \cdots & \frac{\partial {{f}_{n}}}{\partial {{x}_{n}}} \\ \end{array} \right]. $$

Assume *x* ^∗^ is an equilibrium point, i.e., *f*(*x* ^∗^)=0. *J* ^∗^ is the Jacobian matrix evaluated at *x* ^∗^. The equilibrium point *x* ^∗^ is stable if all the eigenvalues of the characteristic equation of *J* ^∗^ have negative real parts [22].

#### Sensitivity analysis

We conduct sensitivity analysis to investigate the significance of model parameters. Sensitivity analysis studies the uncertainty of the output of a model caused by the uncertainty of the inputs of the model [23, 24]. Assume the output (*Y*) of a model can be represented as the following equation:

$$Y=f({{x}_{1}},{{x}_{2}},{\ldots},{{x}_{n}}) $$

where *x*_*i*_ (*i*=1,2,…*n*) represent the input variables or factors. Each time, we analyze the sensitivity of one factor (or parameter) by assigning a perturbation term *ε* *β* to the factor, e.g., *x*_*i*_, where *ε* denotes the disturbance intensity and *β* is a random number that satisfies the uniform distribution between [-1, 1]. We randomly sample values from the interval [*x*_*i*_−*ε*,*x*_*i*_+*ε*] and then compute the mean and variance of the output as follows:

$$\begin{aligned} & \bar{Y}=\frac{1}{K}\sum\limits_{k=1}^{K}{{{Y}_{k}}}, \\ & {{S}^{2}}=\sum\limits_{k=1}^{K}{{{({{Y}_{k}}-\bar{Y})}^{2}}}/(K-1), \\ \end{aligned} $$

where *Y*_*k*_ (*k*=1,2,…,*K*) are values of the output *Y*.

## Results and discussion

In what follows, we present analysis and simulation results for the model.

### Local stability analysis

System (1) gives the dynamic equations of *NC* cells, which is impossible to be analyzed for a big *NC* in theory. However, due to the locality of the Delta-Notch interaction, we can still obtain valuable insight into the whole system by only considering two cells to explore their dynamic behavior such as equilibria and stability [25–27] from the theoretical point of view. The mathematical model of two cells is given as follows:

2 $$\begin{array}{@{}rcl@{}} {{\left\{ \begin{aligned} & \frac{d{{D}_{1}}}{dt}=\frac{\lambda }{1+\Delta \cdot {{A}_{1}}}-{{d}_{1}}{{D}_{1}}-{{f}_{1}}\cdot {{D}_{1}}, \\ & \frac{d{{N}_{1}}}{dt}={{\lambda }_{N}}-{{d}_{2}}{{N}_{1}}+{{f}_{2}}\cdot {{D}_{2}}-\frac{a{{N}_{1}}}{b{{D}_{1}}+{{N}_{1}}}, \\ & \frac{d{{A}_{1}}}{dt}=-{{d}_{3}}{{A}_{1}}+\frac{a{{N}_{1}}}{b{{D}_{1}}+{{N}_{1}}}, \\ & \frac{d{{D}_{2}}}{dt}=\frac{\lambda }{1+\Delta \cdot {{A}_{2}}}-{{d}_{1}}{{D}_{2}}-{{f}_{1}}\cdot {{D}_{2}}, \\ & \frac{d{{N}_{2}}}{dt}={{\lambda }_{N}}-{{d}_{2}}{{N}_{2}}+{{f}_{2}}\cdot {{D}_{1}}-\frac{a{{N}_{2}}}{b{{D}_{2}}+{{N}_{2}}}, \\ & \frac{d{{A}_{2}}}{dt}=-{{d}_{3}}{{A}_{2}}+\frac{a{{N}_{2}}}{b{{D}_{2}}+{{N}_{2}}}. \\ \end{aligned} \right.}} \end{array} $$

In the following, we will take *λ* (the production rate of Delta proteins) as an example to study the equilibria and stability of System (2).

(1) When *λ*=0, which corresponds to mis-expression of Delta, we obtain the equilibrium

$${{E}_{0}}=\left(D_{1}^{0},N_{1}^{0},A_{1}^{0},D_{2}^{0},N_{2}^{0},A_{2}^{0}\right), $$

where $D_{1}^{0}=0$, $N_{1}^{0}=\frac {{{\lambda }_{N}}-a}{{{d}_{2}}}$, $A_{1}^{0}=\frac {a}{{{d}_{3}}}$, $D_{2}^{0}=0$, $N_{2}^{0}=\frac {{{\lambda }_{N}}-a}{{{d}_{2}}}$ and $A_{2}^{0}=\frac {a}{{{d}_{3}}}$.

(2) When *λ*>0, which corresponds to normal or over-expression of Delta, we obtain the equilibrium

$${{E}_{1}}=\left(D_{1}^{1},N_{1}^{1},A_{1}^{1},D_{2}^{1},N_{2}^{1},A_{2}^{1}\right), $$

where $D_{1}^{1}=\frac {\lambda }{\left (1+\Delta A_{1}^{1}\right)\left ({{d}_{1}}+{{f}_{1}}\right)}$, $N_{1}^{1}=\frac {b{{d}_{3}}\lambda A_{1}^{1}}{\left (1+\Delta A_{1}^{1}\right)\left ({{d}_{1}}+{{f}_{1}}\right)\left (a-{{d}_{3}}A_{1}^{1}\right)}$, $D_{2}^{1}=\frac {\lambda }{\left (1+\Delta A_{1}^{1}\right)\left ({{d}_{1}}+{{f}_{1}}\right)}$, $N_{2}^{1}=\frac {b{{d}_{3}}\lambda A_{1}^{1}}{\left (1+\Delta A_{1}^{1}\right)\left ({{d}_{1}}+{{f}_{1}}\right)\left (a-{{d}_{3}}A_{1}^{1}\right)}$ and $A_{2}^{1}=A_{1}^{1}$. $A_{1}^{1}$ is the solution of

3 $$ \begin{aligned} & -d_{3}^{2}\left({{d}_{1}}+{{f}_{1}}\right)\Delta {{\left(A_{1}^{1}\right)}^{3}}+\left[{{d}_{3}}\left({{d}_{1}}+{{f}_{1}}\right)\left(a\Delta -{{d}_{3}}\right) +\Delta {{d}_{3}}{{\lambda }_{N}}\left({{d}_{1}}+{{f}_{1}}\right)\right]{{\left(A_{1}^{1}\right)}^{2}} \\ & +\left[a{{d}_{3}}\left({{d}_{1}}+{{f}_{1}}\right)-{{\lambda }_{N}}\left({{d}_{1}}+{{f}_{1}}\right)\left(a\Delta -{{d}_{3}}\right)+{{d}_{3}}{{f}_{2}}\lambda +b{{d}_{2}}{{d}_{3}}\lambda \right]A_{1}^{1} \\ & -a{{\lambda }_{N}}\left({{d}_{1}}+{{f}_{1}}\right)-a{{f}_{2}}\lambda =0. \\ \end{aligned}  $$

Now, let

$$\begin{aligned} & {{m}_{1}}=-d_{3}^{2}({{d}_{1}}+{{f}_{1}})\Delta, \\ & {{m}_{2}}={{d}_{3}}({{d}_{1}}+{{f}_{1}})(a\Delta -{{d}_{3}})+\Delta {{d}_{3}}{{\lambda }_{N}}({{d}_{1}}+{{f}_{1}}), \\ & {{m}_{3}}=a{{d}_{3}}({{d}_{1}}+{{f}_{1}})-{{\lambda }_{N}}({{d}_{1}}+{{f}_{1}})(a\Delta -{{d}_{3}}) +{{d}_{3}}{{f}_{2}}\lambda +b{{d}_{2}}{{d}_{3}}\lambda, \\ & {{m}_{4}}=-a{{\lambda }_{N}}({{d}_{1}}+{{f}_{1}})-a{{f}_{2}}\lambda. \\ \end{aligned} $$

Then Eq. (3) becomes

$${{m}_{1}}{{\left(A_{1}^{1}\right)}^{3}}+{{m}_{2}}{{\left(A_{1}^{1}\right)}^{2}}+{{m}_{3}}A_{1}^{1}+{{m}_{4}}=0. $$

Because ${{m}_{1}}=-d_{3}^{2}({{d}_{1}}+{{f}_{1}})\Delta \ne 0$, we further obtain

$${{\left(A_{1}^{1}\right)}^{3}}+\frac{{{m}_{2}}}{{{m}_{1}}}{{\left(A_{1}^{1}\right)}^{2}}+\frac{{{m}_{3}}}{{{m}_{1}}}A_{1}^{1}+\frac{{{m}_{4}}}{{{m}_{1}}}=0. $$

Thus, we have

$$\begin{aligned} & A_{1}^{1}= \\ & \sqrt[3]{-\frac{\frac{2m_{2}^{3}}{27m_{1}^{3}}-\frac{{{m}_{2}}{{m}_{3}}}{3m_{1}^{2}}+\frac{{{m}_{4}}}{{{m}_{1}}}}{2}\,+\,\sqrt{{{\left(\! \frac{\frac{2m_{2}^{3}}{27m_{1}^{3}}-\frac{{{m}_{2}}{{m}_{3}}}{3m_{1}^{2}}+\frac{{{m}_{4}}}{{{m}_{1}}}}{2} \!\right)}^{2}}\,+\,{{\left(\frac{-\frac{m_{2}^{2}}{3m_{1}^{2}}+\frac{{{m}_{3}}}{{{m}_{1}}}}{3} \right)}^{3}}}} \\ & +\sqrt[3]{-\frac{\frac{2m_{2}^{3}}{27m_{1}^{3}}-\frac{{{m}_{2}}{{m}_{3}}}{3m_{1}^{2}}+\frac{{{m}_{4}}}{{{m}_{1}}}}{2}\,-\,\sqrt{{{\left(\!\! \frac{\frac{2m_{2}^{3}}{27m_{1}^{3}}-\frac{{{m}_{2}}{{m}_{3}}}{3m_{1}^{2}}+\frac{{{m}_{4}}}{{{m}_{1}}}}{2} \!\!\right)}^{2}}\,+\,{{\left(\!\! \frac{-\frac{m_{2}^{2}}{3m_{1}^{2}}+\frac{{{m}_{3}}}{{{m}_{1}}}}{3} \!\!\right)}^{3}}}} \\ & -\frac{{{m}_{2}}}{3{{m}_{1}}}. \\ \end{aligned} $$

Please note that the equilibria of System (2) we obtain above mean that the system will converge to a stable state after a period of time.

Now, we further study the stability of System (2) at equilibrium *E*_0_. The Jacobi matrix [27] at *E*_0_ is computed as follows:

$${{J}_{{{E}_{0}}}}=\left[ \begin{array}{cccccc} -{{d}_{1}}-{{f}_{1}} & 0 & 0 & 0 & 0 & 0 \\ \frac{abN_{1}^{0}}{{{\left(bD_{1}^{0}+N_{1}^{0}\right)}^{2}}} & -{{d}_{2}}-\frac{abD_{1}^{0}}{{{\left(bD_{1}^{0}+N_{1}^{0}\right)}^{2}}} & 0 & {{f}_{2}} & 0 & 0\\ \frac{-abN_{1}^{0}}{{{\left(bD_{1}^{0}+N_{1}^{0}\right)}^{2}}} & \frac{abD_{1}^{0}}{{{\left(bD_{1}^{0}+N_{1}^{0}\right)}^{2}}} & -{{d}_{3}} & 0 & 0 & 0 \\ 0 & 0 & 0 & -{{d}_{1}}-{{f}_{1}} & 0 & 0 \\ {{f}_{2}} & 0 & 0 & \frac{abN_{2}^{0}}{{{\left(bD_{2}^{0}+N_{2}^{0}\right)}^{2}}} & -{{d}_{2}}-\frac{abD_{2}^{0}}{{{\left(bD_{2}^{0}+N_{2}^{0}\right)}^{2}}} & 0 \\ 0 & 0 & 0 & \frac{-abN_{2}^{0}}{{{\left(bD_{2}^{0}+N_{2}^{0}\right)}^{2}}} & \frac{abD_{2}^{0}}{{{\left(bD_{2}^{0}+N_{2}^{0}\right)}^{2}}} & -{{d}_{3}} \\ \end{array} \right]. $$

Assume *S* is the eigenvalue of the characteristic equation $\phantom {\dot {i}\!}|{{J}_{{{E}_{0}}}}|$. Then the characteristic equation is calculated as follows [27]:

$$\left| {{J}_{{{E}_{0}}}} \right|=\left| \begin{array}{cccccc} S+{{d}_{1}}+{{f}_{1}} & 0 & 0 & 0 & 0 & 0 \\ \frac{-abN_{1}^{0}}{{{\left(bD_{1}^{0}+N_{1}^{0}\right)}^{2}}} & S+{{d}_{2}}+\frac{abD_{1}^{0}}{{{\left(bD_{1}^{0}+N_{1}^{0}\right)}^{2}}} & 0 & -{{f}_{2}} & 0 & 0 \\ \frac{abN_{1}^{0}}{{{\left(bD_{1}^{0}+N_{1}^{0}\right)}^{2}}} & \frac{-abD_{1}^{0}}{{{\left(bD_{1}^{0}+N_{1}^{0}\right)}^{2}}} & S+{{d}_{3}} & 0 & 0 & 0 \\ 0 & 0 & 0 & S+{{d}_{1}}+{{f}_{1}} & 0 & 0 \\ -{{f}_{2}} & 0 & 0 & \frac{-abN_{2}^{0}}{{{\left(bD_{2}^{0}+N_{2}^{0}\right)}^{2}}} & S+{{d}_{2}}+\frac{abD_{2}^{0}}{{{\left(bD_{2}^{0}+N_{2}^{0}\right)}^{2}}} & 0 \\ 0 & 0 & 0 & \frac{abN_{2}^{0}}{{{\left(bD_{2}^{0}+N_{2}^{0}\right)}^{2}}} & \frac{-abD_{2}^{0}}{{{\left(bD_{2}^{0}+N_{2}^{0}\right)}^{2}}} & S+{{d}_{3}} \\ \end{array} \right|. $$

After some intermediate computation steps, we have

4 $$ | {{J}_{{{E}_{0}}}}|={{(S+{{d}_{1}}+{{f}_{1}})}^{2}}{{(S+{{d}_{2}})}^{2}}{{(S+{{d}_{3}})}^{2}}  $$

It is obvious that all of the six eigenvalues of Eq. (4) are negative, which gives the following conclusion.

#### **Lemma 1**

The equilibrium *E*_0_ is locally asymptotically stable.

**Fig. 6:**
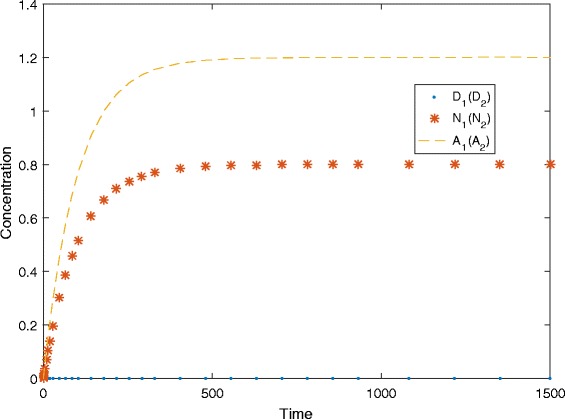
A simulation plot at *λ*=0. In this plot, the simulation traces of *D*_1_ (*N*_1_, *A*_1_) and *D*_2_ (*N*_2_, *A*_2_) overlap

For example, a simulation in this case is given in Fig. 6, with the following parameter values: *λ*_*N*_=0.02, *d*_1_=*d*_2_=*d*_3_=0.01, *f*_1_=*f*_2_=0.665, *a*=0.012, *b*=69, *Δ*=10^6^ and *λ*=0.

Next, we investigate the stability of the equilibrium *E*_1_. The Jacobi matrix at *E*_1_ is given as follows:

$${{J}_{{{E}_{1}}}}=\left[ \begin{array}{cccccc} -{{d}_{1}}-{{f}_{1}} & 0 & \frac{-\Delta \lambda }{{{\left(1+\Delta A_{1}^{1}\right)}^{2}}} & 0 & 0 & 0 \\ \frac{abN_{1}^{1}}{{{\left(bD_{1}^{1}+N_{1}^{1}\right)}^{2}}} & -{{d}_{2}}-\frac{abD_{1}^{1}}{{{\left(bD_{1}^{1}+N_{1}^{1}\right)}^{2}}} & 0 & {{f}_{2}} & 0 & 0 \\ \frac{-abN_{1}^{1}}{{{\left(bD_{1}^{1}+N_{1}^{1}\right)}^{2}}} & \frac{abD_{1}^{0}}{{{\left(bD_{1}^{1}+N_{1}^{1}\right)}^{2}}} & -{{d}_{3}} & 0 & 0 & 0 \\ 0 & 0 & 0 & -{{d}_{1}}-{{f}_{1}} & 0 & \frac{-\Delta \lambda }{{{\left(1+\Delta A_{2}^{1}\right)}^{2}}} \\ {{f}_{2}} & 0 & 0 & \frac{abN_{2}^{1}}{{{\left(bD_{2}^{1}+N_{2}^{1}\right)}^{2}}} & -{{d}_{2}}-\frac{abD_{2}^{1}}{{{\left(bD_{2}^{1}+N_{2}^{1}\right)}^{2}}} & 0 \\ 0 & 0 & 0 & \frac{-abN_{2}^{1}}{{{\left(bD_{2}^{1}+N_{2}^{1}\right)}^{2}}} & \frac{abD_{2}^{1}}{{{\left(bD_{2}^{1}+N_{2}^{1}\right)}^{2}}} & -{{d}_{3}} \\ \end{array} \right].$$

The characteristic equation is:

$$\begin{aligned} & \left| {{J}_{{{E}_{1}}}} \right|= \\ & \left| \begin{array}{cccccc} S+{{d}_{1}}+{{f}_{1}} & 0 & \frac{\Delta \lambda }{{{\left(1+\Delta A_{1}^{1}\right)}^{2}}} & 0 & 0 & 0 \\ \frac{-abN_{1}^{1}}{{{\left(bD_{1}^{1}+N_{1}^{1}\right)}^{2}}} & S+{{d}_{2}}+\frac{abD_{1}^{1}}{{{\left(bD_{1}^{1}+N_{1}^{1}\right)}^{2}}} & 0 & -{{f}_{2}} & 0 & 0 \\ \frac{abN_{1}^{1}}{{{\left(bD_{1}^{1}+N_{1}^{1}\right)}^{2}}} & \frac{-abD_{1}^{1}}{{{\left(bD_{1}^{1}+N_{1}^{1}\right)}^{2}}} & S+{{d}_{3}} & 0 & 0 & 0 \\ 0 & 0 & 0 & S+{{d}_{1}}+{{f}_{1}} & 0 & \frac{\Delta \lambda }{{{\left(1+\Delta A_{2}^{1}\right)}^{2}}} \\ -{{f}_{2}} & 0 & 0 & \frac{-abN_{2}^{1}}{{{\left(bD_{2}^{1}+N_{2}^{1}\right)}^{2}}} & S+{{d}_{2}}+\frac{abD_{2}^{1}}{{{\left(bD_{2}^{1}+N_{2}^{1}\right)}^{2}}} & 0 \\ 0 & 0 & 0 & \frac{abN_{2}^{1}}{{{\left(bD_{2}^{1}+N_{2}^{1}\right)}^{2}}} & \frac{-abD_{2}^{1}}{{{\left(bD_{2}^{1}+N_{2}^{1}\right)}^{2}}} & S+{{d}_{3}} \\ \end{array} \right|. \end{aligned} $$

Let

$${{F}_{1}}=\left[ \begin{array}{ccc} S+{{d}_{1}}+{{f}_{1}} & 0 & \frac{\Delta \lambda }{{{\left(1+\Delta A_{1}^{1}\right)}^{2}}} \\ \frac{-abN_{1}^{1}}{{{\left(bD_{1}^{1}+N_{1}^{1}\right)}^{2}}} & S+{{d}_{2}}+\frac{abD_{1}^{1}}{{{\left(bD_{1}^{1}+N_{1}^{1}\right)}^{2}}} & 0 \\ \frac{abN_{1}^{1}}{{{\left(bD_{1}^{1}+N_{1}^{1}\right)}^{2}}} & \frac{-abD_{1}^{1}}{{{\left(bD_{1}^{1}+N_{1}^{1}\right)}^{2}}} & S+{{d}_{3}} \\ \end{array} \right], {{F}_{2}}=\left[ \begin{array}{ccc} 0 & 0 & 0 \\ -{{f}_{2}} & 0 & 0 \\ 0 & 0 & 0 \\ \end{array} \right]$$

and

$${{F}_{3}}=\left[ \begin{array}{ccc} S+{{d}_{1}}+{{f}_{1}} & 0 & \frac{\Delta \lambda }{{{\left(1+\Delta A_{2}^{1}\right)}^{2}}} \\ \frac{-abN_{1}^{1}}{{{\left(bD_{2}^{1}+N_{2}^{1}\right)}^{2}}} & S+{{d}_{2}}+\frac{abD_{2}^{1}}{{{\left(bD_{2}^{1}+N_{2}^{1}\right)}^{2}}} & 0 \\ \frac{abN_{2}^{1}}{{{\left(bD_{2}^{1}+N_{2}^{1}\right)}^{2}}} & \frac{-abD_{1}^{1}}{{{\left(bD_{2}^{1}+N_{2}^{1}\right)}^{2}}} & S+{{d}_{3}} \\ \end{array} \right]. $$

Then we obtain

$$\left| {{J}_{{{E}_{1}}}} \right|=\left| \begin{array}{cc} {{F}_{1}} & {{F}_{2}} \\ {{F}_{2}} & {{F}_{3}} \\ \end{array} \right|. $$

As $D_{1}^{1}=D_{2}^{1},N_{1}^{1}=N_{2}^{1},A_{1}^{1}=A_{2}^{1}$, we have *F*_1_=*F*_3_. We further have

5 $$ \left| {{J}_{{{E}_{1}}}} \right|=\left| \begin{array}{cc} {{F}_{1}} & {{F}_{2}} \\ {{F}_{2}} & {{F}_{1}} \\ \end{array} \right|=\left| {{F}_{1}}+{{F}_{2}} \right|\cdot \left| {{F}_{1}}-{{F}_{2}}\right|.  $$

$$\begin{aligned} \left| {{F}_{1}}+{{F}_{2}} \right|&=\left| \begin{array}{ccc} S+{{d}_{1}}+{{f}_{1}} & 0 & \frac{\Delta \lambda }{{{(1+\Delta A_{1}^{1})}^{2}}} \\ \frac{-abN_{1}^{1}}{{{(bD_{1}^{1}+N_{1}^{1})}^{2}}}-{{f}_{2}} & S+{{d}_{2}}+\frac{abD_{1}^{1}}{{{(bD_{1}^{1}+N_{1}^{1})}^{2}}} & 0 \\ \frac{abN_{1}^{1}}{{{(bD_{1}^{1}+N_{1}^{1})}^{2}}} & \frac{-abD_{1}^{1}}{{{(bD_{1}^{1}+N_{1}^{1})}^{2}}} & S+{{d}_{3}} \\ \end{array} \right| \\ & ={{S}^{3}}+{{M}_{1}}{{S}^{2}}+{{M}_{2}}S+{{M}_{3}}, \\ \end{aligned} $$

where

$${} {{\begin{aligned} {{M}_{1}}&= {{d}_{2}}+\frac{abD_{1}^{1}}{{{\left(bD_{1}^{1}+N_{1}^{1}\right)}^{2}}}+{{d}_{1}}+{{f}_{1}}+{{d}_{3}}, \\ {{M}_{2}}&= \left({{d}_{1}}+{{f}_{1}}\right){{d}_{3}}+\left({{d}_{1}}+{{f}_{1}}+{{d}_{3}}\right)\left({{d}_{2}}+\frac{abD_{1}^{1}}{{{\left(bD_{1}^{1}+N_{1}^{1}\right)}^{2}}}\right) \\&\quad+\frac{\Delta \lambda }{{{\left(1+\Delta A_{1}^{1}\right)}^{2}}}\cdot \frac{abN_{1}^{1}}{{{\left(bD_{1}^{1}+N_{1}^{1}\right)}^{2}}}, \\ {{M}_{3}}&= \left({{d}_{1}}+{{f}_{1}}\right){{d}_{3}}\left({{d}_{2}}+\frac{abD_{1}^{1}}{{{\left(bD_{1}^{1}+N_{1}^{1}\right)}^{2}}}\right)\\&\quad +\frac{\Delta \lambda }{{{\left(1+\Delta A_{1}^{1}\right)}^{2}}}\left[\frac{abN_{1}^{1}}{{{\left(bD_{1}^{1}+N_{1}^{1}\right)}^{2}}} \left({{d}_{2}}+\frac{abD_{1}^{1}}{{{\left(bD_{1}^{1}+N_{1}^{1}\right)}^{2}}}\right) \right.\\ &\left.\quad+\left(\frac{abN_{1}^{1}}{{{\left(bD_{1}^{1}+N_{1}^{1}\right)}^{2}}}+{{f}_{2}}\right)\frac{abD_{1}^{1}}{{{\left(bD_{1}^{1}+N_{1}^{1}\right)}^{2}}}\right]. \\ \end{aligned}}} $$

Similarly, we have

$$\left| {{F}_{1}}-{{F}_{2}} \right|={{S}^{3}}+{{M}_{1}}{{S}^{2}}+{{M}_{2}}S+{{{M}'}_{3}}, $$

where

$${} {{\begin{aligned} {{{{M}'}}_{3}}=& \left({{d}_{1}}+{{f}_{1}}\right){{d}_{3}}\left({{d}_{2}}+\frac{abD_{1}^{1}}{{{\left(bD_{1}^{1}+N_{1}^{1}\right)}^{2}}}\right)\\ & \quad +\frac{\Delta \lambda }{{{\left(1+\Delta A_{1}^{1}\right)}^{2}}}\left[\frac{abN_{1}^{1}}{{{\left(bD_{1}^{1}+N_{1}^{1}\right)}^{2}}} \left({{d}_{2}}+\frac{abD_{1}^{1}}{{{\left(bD_{1}^{1}+N_{1}^{1}\right)}^{2}}}\right) \right.\\ & \left.+\left(\frac{abN_{1}^{1}}{{{\left(bD_{1}^{1}+N_{1}^{1}\right)}^{2}}}-{{f}_{2}}\right)\frac{abD_{1}^{1}}{{{\left(bD_{1}^{1}+N_{1}^{1}\right)}^{2}}}\right]. \\ \end{aligned}}} $$

Therefore, Eq. (5) becomes

6 $$\begin{array}{@{}rcl@{}} {{\begin{aligned} \left| {{J}_{{{E}_{1}}}} \right|& =\left| {{F}_{1}}+{{F}_{2}} \right|\cdot \left| {{F}_{1}}-{{F}_{2}} \right| \\ & =\left({{S}^{3}}+{{M}_{1}}{{S}^{2}}+{{M}_{2}}S+{{M}_{3}}\right)\left({{S}^{3}}+{{M}_{1}}{{S}^{2}}+{{M}_{2}}S+{{{{M}'}}_{3}}\right). \\ \end{aligned}}} \end{array} $$

Using the Routh-Hurwitz criterion for Eq. (6), we obtain

$${{\Delta }_{1}}\equiv 1>0,\ {{\Delta }_{2}}\equiv \left| \begin{array}{cc} {{M}_{1}} & 1 \\ {{M}_{3}} & {{M}_{2}} \\ \end{array} \right|>0,\ $$

and

$$\Delta_{1}^{'}\equiv 1>0,\ \Delta_{2}^{'}\equiv \left| \begin{array}{cc} {{M}_{1}} & 1 \\ {{{{M}'}}_{3}} & {{M}_{2}} \\ \end{array} \right|>0. $$

Namely, *M*_1_ *M*_2_−*M*_3_>0, and *M*_1_ *M*_2_−*M* ^′^_3_>0. Because *M*_3_>*M* ^′^_3_, we only need the condition *M*_1_ *M*_2_−*M*_3_>0 to be satisfied. That is, if $\frac {{{M}_{3}}}{{{M}_{1}}{{M}_{2}}}<1$, the eigenvalues of |*F*_1_+*F*_2_|=0 and |*F*_1_−*F*_2_|=0 are negative; thus, *E*_1_ is locally stable.

#### **Lemma 2**

If $\frac {{{M}_{3}}}{{{M}_{1}}{{M}_{2}}}<1$, the equilibrium *E*_1_ is locally asymptotically stable; if$\frac {{{M}_{3}}}{{{M}_{1}}{{M}_{2}}}>1$, it is unstable.

**Fig. 7:**
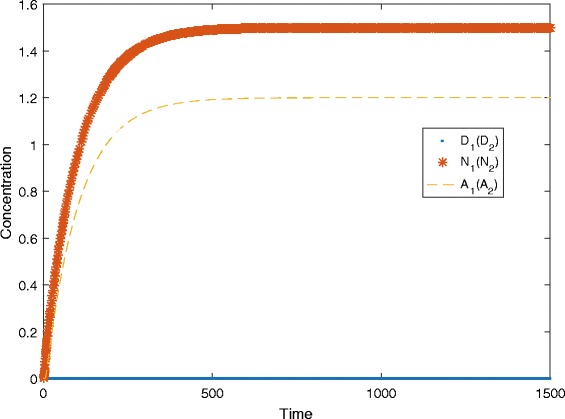
A simulation plot at *λ*>0. In this plot, the simulation traces of *D*_1_ (*N*_1_, *A*_1_) and *D*_2_ (*N*_2_, *A*_2_) overlap

For example, a simulation in this case is given in Fig. 7 with the following parameter values: *λ*_*N*_=0.027, *d*_1_=*d*_2_=*d*_3_=0.01, *f*_1_=*f*_2_=0.665, *a*=0.012, *b*=69, *Δ*=10^6^ and *λ*=35.

Please note that the equilibria and stability analysis above show that the model would reach an equilibrium in either case of *λ*, which corresponds to that the system would result in a stable pattern in either normal or mutant conditions. If there are small disturbances of parameters (e.g., small environmental noises), the system would always converge to a stable state after a period of time when *λ*=0 according to Lemma 1, but the system would converge to a stable state for *λ*>0 when the stability conditions in Lemma 2 are satisfied. Besides, we will also use Lemma 2 to carefully choose appropriate parameter values for preserving the stability of the system. This is expected to be shown in the the boundary formation model of the *Drosophila* large intestine.

### Deterministic simulation results

However, it is impossible to analyze the model above with a large number of cells in theory, e.g., a model with 60 cells would result in 180 dimensional ordinary differential equations. In this section, we will explore the model using numerical simulation.

The simulation starts from a prepattern of Delta expression in normal large intestines, that is, Delta is expressed only in the ventral region [13]. For this, we set the production rate of the Delta level as follows: 0 for cells at the first three rows (dorsal cells) and *λ* (greater than 0) for cells at the other rows (ventral cells). We set the time span to [0, 1500]. The parameter values are given in Table 1; except *λ* and *λ*_*N*_, other parameter values are fixed throughout the paper. We use the same setting for all the following simulations.

**Table 1:** Parameter values of the model

Parameter	Value
*d*_1_	0.01
*d*_2_	0.01
*d*_3_	0.01
*f*_1_	0.665
*f*_2_	0.665
*a*	0.012
*b*	69
*Δ*	10^6^
*λ*	[0, 12]
*λ*_*N*_	[0. 0.1]

Except *λ* and *λ*_*N*_, the other parameter values are used throughout the paper

Besides, we determine a boundary cell using the following rule. If the concentration of the active Notch proteins (*A*) in a cell is equal to or greater than 1.0, we regard the cell as a boundary cell.

**Fig. 8:**
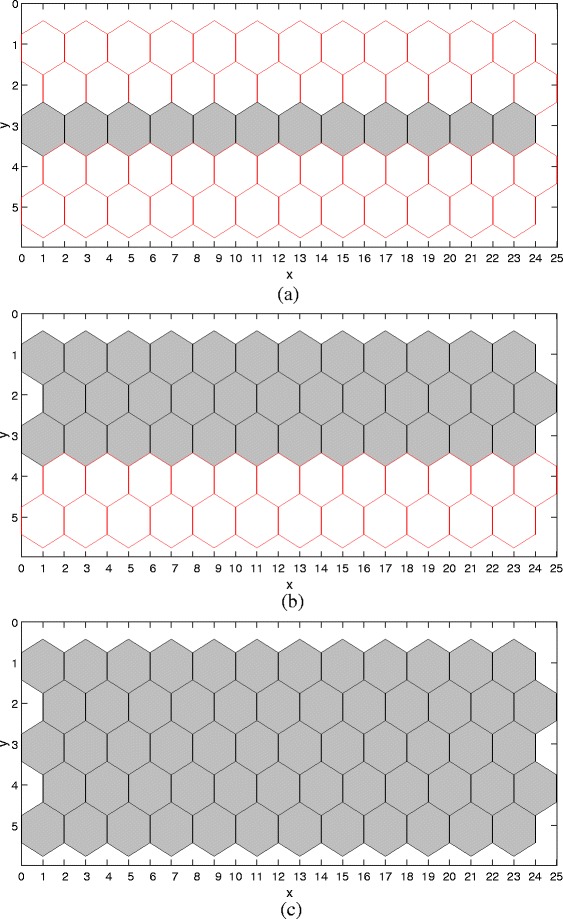
Deterministic simulation results. **a** Wild-type: *λ*_*N*_=0 and *λ*=9, **b** over-expression of Notch: *λ*_*N*_=0.008 and *λ*=9, and, **c** over-expression of Notch: *λ*_*N*_=0.1 and *λ*=9

In the following, we run the model given in System (1) with the parameter values given in Table 1 to obtain deterministic simulation results. We first validate the ability of our model to reproduce the wild-type result observed in the wet lab. We run simulation and obtain the result in the wild-type condition, illustrated in Fig. 8a. We can see that a single strand of boundary cells that are abutting the ventral region is produced, which is consistent with the experimental observation (corresponding to Scenario 1 and Fig. 3a). We also run the model in other conditions such as over-expression of Notch, and obtain simulation results (see Fig. 8b-c), which are also consistent with experimental observations (see Fig. 3 b1-c2). That is, our model has the ability to reproduce the experimental observations obtained in the wet lab.

### Phenotype change due to the change of Delta or Notch expression

During the boundary formation in the *Drosophila* large intestine, different Delta or Notch expression (normal, mis-expression or over-expression) may result in different boundary cell distributions (phenotypes) [1]. In our model, we try to incorporate all these phenotypes reported so far. As shown in System (1), Delta or Notch expression is controlled by the rates *λ* and *λ*_*N*_, respectively. Therefore, we need to map different Delta and Notch expression observed in experiments to different values of *λ* and *λ*_*N*_ in the model.

To do this, we explore the phenotype change due to the change of Delta or Notch expression in their parameter space and then determine what parameter value results in what kind of phenotype. As well, we cannot adopt a theoretical analysis due to the large number of equations involved, and instead, we still use numerical simulation. Besides, each time we tune only one parameter, either *λ* or *λ*_*N*_, by fixing the other.

#### Phenotype change due to the change of Delta expression

By fixing *λ*_*N*_=0.0005, we gradually increase *λ* from 0 to 12 with a step 0.1 and then run simulation to obtain the simulation result, given in Table 2.

**Table 2:** Phenotype change due to the change of Delta expression

Expression	Phenotype
12≥*λ*>0.2	At the first three rows are boundary cells
0≤*λ*≤0.2	At all five rows are boundary cells

By fixing *λ*_*N*_=0.0005, and gradually increasing *λ* from 0 to 12 with a step 0.1, we obtain the phenotype change with the change of Delta expression

From the table above, we can see when *λ*>0.2, the first three rows are stable boundary cells, while when *λ*≤0.2, all the five rows are boundary cells. This means there are only two phenotypes appearing due to the change of Delta expression.

#### Phenotype change due to the change of Notch expression

By fixing *λ*=9, we gradually increase *λ*_*N*_ from 0 to 0.1 with a small step 0.0001 and then run simulation to obtain the simulation result, given in Table 3.

**Table 3:** Phenotype change due to the change of Notch expression

Expression	Phenotype
0≤*λ*_*N*_<0.0001	Only at the third row are boundary cells
0.0001≤*λ*_*N*_<0.0098	At the first three rows are boundary cells
0.0098≤*λ*_*N*_<0.01	At the first four rows are boundary cells
0.01≤*λ*_*N*_≤0.1	At all five rows are boundary cells

By fixing *λ*=9, and gradually increasing *λ*_*N*_ from 0 to 0.1 with a small step 0.0001, we obtain the phenotype change with the change of Notch expression

Table 3 shows with the increase of *λ*_*N*_, the number of boundary cells increases, which results in a couple of different phenotypes. Besides, we should notice that a tiny parameter change would result in a big change of the number of boundary cells, so we can deduce that parameter *λ*_*N*_ is very sensitive.

According to the analysis result above, we finally map different Delta and Notch expression observed in experiments to different values of *λ* and *λ*_*N*_ in the model. Besides, we can reproduce all known biological phenotypes we have found by combining different values of *λ* and *λ*_*N*_.

### Simulation results with noises of Notch expression

As described in [1], the boundary formation of the *Drosophila* large intestine is greatly affected by temperature; the random effect of temperature may cause fluctuations of gene expression levels, resulting in different phenotypes, in which boundary cells may randomly be distributed in the dorsal and ventral domains. In this section, we will discuss how to use our model to obtain these random phenotypes.

From Tables 2 and 3, we know that only *λ*_*N*_ is sensitive to System (1), so we only add noises to *λ*_*N*_. Then, System (1) with a random noise becomes the following form:

7 $${} \left\{ \begin{aligned} & \frac{d{{D}_{i}}}{dt}=\frac{\lambda }{1+\Delta \cdot {{A}_{i}}}-{{d}_{1}}{{D}_{i}}-\sum\limits_{NG(i)}{{{f}_{1}}\cdot {{D}_{i}}}, \\ & \frac{d{{N}_{i}}}{dt}=({{\lambda }_{N}}+e)-{{d}_{2}}{{N}_{i}}+\sum\limits_{j\in NG(i)}{{{f}_{2}}\cdot {{D}_{j}}}-\frac{a{{N}_{i}}}{b{{D}_{i}}+{{N}_{i}}}, \\ & \frac{d{{A}_{i}}}{dt}=-{{d}_{3}}{{A}_{i}}+\frac{a{{N}_{i}}}{b{{D}_{i}}+{{N}_{i}}}, \\ \end{aligned} \right.  $$

where *e* is a small noise.

**Fig. 9:**
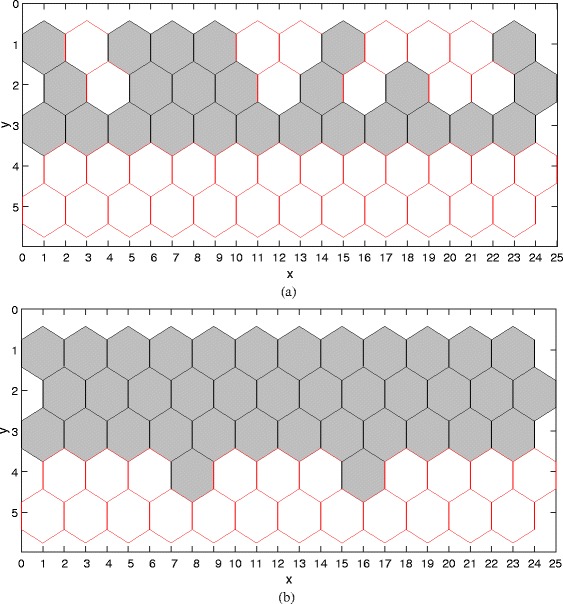
Two random simulation runs at *λ*_*N*_=0.0005 and *λ*=0.3. The noises are randomly sampled from [−5·10^−4^,5·10^−4^]

Running this model with different *λ*_*N*_ values, we can obtain different random phenotypes. Figure 9 illustrates some phenotypes. So far, we have illustrated that our model reproduces all the phenotypes corresponding to those four scenarios we give above.

### Perturbation influences of binding and inhibition parameters on boundary formation

During the development of the *Drosophila* large intestine, there are many environmental factors or noises, which may vary and even heavily affect the boundary formation. To make the model more realistic, we need to consider noises into the model, e.g., adding a random term to each parameter of interest, and analyze the model in noisy conditions. In the section above, we have discussed the random effects of Notch expression on the boundary formation. But in this section, we will further analyze the perturbation influences of the binding and inhibition parameters of the Delta-Notch pathway on the boundary formation. That is, we will explore the inhibition parameters, *a* and *b*, and binding parameters, *f*_1_ and *f*_2_.

For these parameters, which ones are of interest? To answer this, we may alternatively find those parameters which have significant influences on the dynamics of the model when they vary. For this, we use sensitivity analysis on the mathematical model to study the influence of parameter perturbation [28]. After that, we can design experiments by considering noises in vivo, and carefully add appropriate noise items to key parameters. We will explore this in the case of over-expression of Notch proteins. For the other cases, we can do the same exploration.

Next, with the parameter setting given in Fig. 8b (of course we can use any other setting), we will explore the influence of parameter perturbation on the boundary formation by changing parameters, *a*, *b*, *f*_1_ and *f*_2_, to different perturbation intensities.

#### Perturbation influence of parameter *a*

First, we explore the perturbation influence of parameter *a*. All the parameters take the values given in Fig. 8b. In order to do this, we add a perturbation term *ε* *β* to parameter *a*, where *ε* denotes the disturbance intensity and *β* is a random number that satisfies the uniform distribution between [-1, 1]. Then, System (1) with a random disturbance becomes the following form:

8 $$ \left\{ \begin{aligned} & \frac{d{{D}_{i}}}{dt}=\frac{\lambda }{1+\Delta \cdot {{A}_{i}}}-{{d}_{1}}{{D}_{i}}-\sum\limits_{NG(i)}{{{f}_{1}}\cdot {{D}_{i}}}, \\ & \frac{d{{N}_{i}}}{dt}={{\lambda }_{N}}-{{d}_{2}}{{N}_{i}}+\sum\limits_{j\in NG(i)}{{{f}_{2}}\cdot {{D}_{j}}}-\frac{(a+\varepsilon {{\beta }_{i}}){{N}_{i}}}{b{{D}_{i}}+{{N}_{i}}}, \\ & \frac{d{{A}_{i}}}{dt}=-{{d}_{3}}{{A}_{i}}+\frac{(a+\varepsilon {{\beta }_{i}}){{N}_{i}}}{b{{D}_{i}}+{{N}_{i}}}. \\ \end{aligned} \right.  $$

**Fig. 10:**
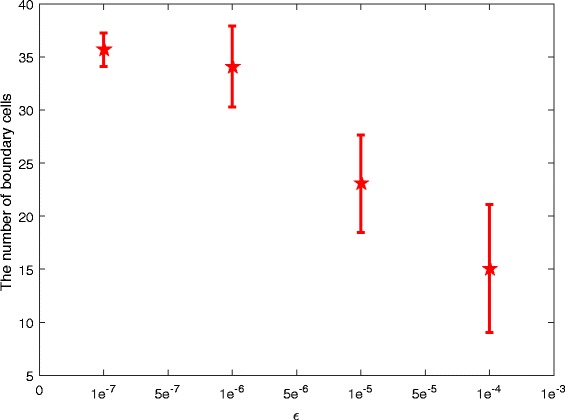
Perturbation influence of parameter *a*. The considered disturbance intensities are: *ε*=10^−7^, *ε*=10^−6^, *ε*=10^−5^ and *ε*=10^−4^, respectively

At the beginning of each simulation, a random noise *ε* *β*_*i*_ for each cell *i* is generated for *a* by sampling *β*_*i*_. By varying *ε* between [0, 0.001] and performing simulation for 50 times at each *ε* level, we compute the mean and standard error of the number of boundary cells. For example, Fig. 10 gives the result when *ε*=10^−7^, *ε*=10^−6^, *ε*=10^−5^ and *ε*=10^−4^, respectively.

From Fig. 10, we can see that a small perturbation of parameter *a* may cause a big change of the number of boundary cells (mean), i.e., the bigger the perturbation intensity is, the less the number of boundary cells is (from 36 boundary cells without perturbation to about 15 boundary cells with a perturbation intensity *ε*=10^−4^). Furthermore, from the error bars, we know that the bigger the perturbation intensity is, the bigger the standard error is, although the smaller the mean is.

#### Perturbation influence of parameter *b*

**Fig. 11:**
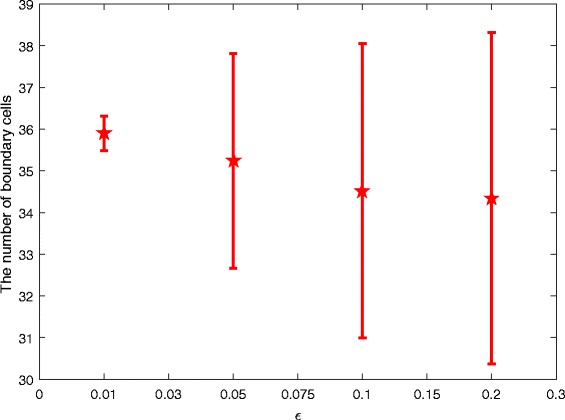
Perturbation influence of parameter *b*. The considered disturbance intensities are: *ε*=0.01, *ε*=0.05, *ε*=0.1 and *ε*=0.2, respectively

We further explore the perturbation influence of parameter *b*. Similarly we add a perturbation term *ε* *β* to *b*, i.e., replacing all *b* with *b*+*ε* *β* in System (1). We also simulate the model in the same way as for parameter *a*, and obtain the mean and standard error of the number of boundary cells when *ε*=0.01, *ε*=0.05, *ε*=0.1 and *ε*=0.2, respectively, illustrated in Fig. 11.

Figure 11 shows that a big perturbation causes a small change (e.g., there are 36 boundary cells without perturbation and around 34 boundary cells with the perturbation intensity *ε*=0.2), which is quite different from the effect of parameter *a*. Therefore, the perturbation influence of parameter *b* is much smaller than that of *a*.

#### Perturbation influence of parameters *f*_1_ and *f*_2_

**Fig. 12:**
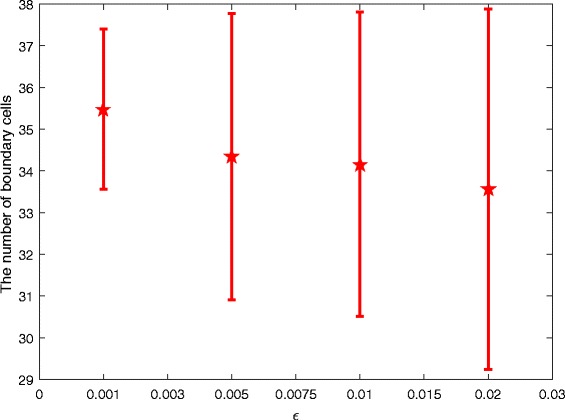
Perturbation influence of parameters *f*_1_ and *f*_2_. The considered disturbance intensities are: *ε*=0.001, *ε*=0.005, *ε*=0.01 and *ε*=0.02, respectively

We further explore the perturbation of *f*_1_ and *f*_2_. For simplicity, we assume *f*_1_=*f*_2_=*f*. Similarly, we add a perturbation term *ε* *β* to *f*_1_ and *f*_2_, i.e., replacing all *f*_1_ and *f*_2_ with *f*+*ε* *β* in System (1). We also simulate the model in the same way as above, and obtain the mean and standard error of the number of boundary cells when *ε*=0.001, *ε*=0.005, *ε*=0.01 and *ε*=0.02, respectively, given in Fig. 12.

From Fig. 12, we can see, similar to parameter *b*, the perturbation of parameter *f* does not have an obvious influence on the number of boundary cells in the given perturbation intensities.

**Fig. 13:**
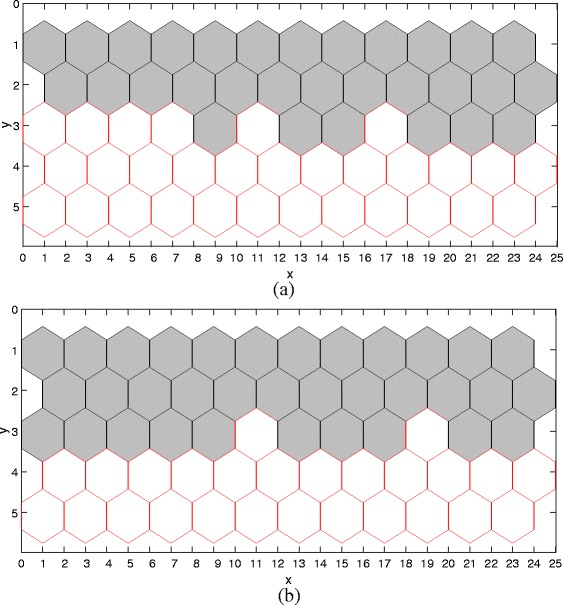
Two simulation runs for parameter *a* with a perturbation intensity *ε*=10^−6^. All the parameters take the values given in Fig. 8b

In conclusion, the analysis results above show that the perturbation of parameter *a* has an obvious influence on the boundary formation, while the other three parameters not. Therefore, when we consider noises in the model, we only add a random term to parameter *a*, and neglect others. That is, we finally obtain a model with random noises, which is given in System (8). With this model, we can make reasonable predictions in noisy conditions by setting the appropriate intensity of the noise according to the analysis result above (see, e.g., Fig. 13).

## Conclusion

In this paper, we give a mathematical model for the Delta-Notch dependent boundary formation in the *Drosophila* large intestine. We aim to use this model to better interpret related biological phenomena and therefore we perform not only theoretical but also simulation analysis to achieve the goal. By combining different analysis techniques, we finally confirm that the model is both mathematically and biologically sound and is sufficient to interpret related experimental findings of the boundary formation in the *Drosophila* large intestine. Moreover, by modulating parameters, our simulation can generate various abberant patterns of boundary cells that have not been reported in biological studies so far. Biologically, these varieties of phenotypes are assumed to be results of variation of gene products in the Delta-Notch pathway. To further verify the validity of our simulation method, we are now trying to develop a biological experimental system to manipulate levels of forced-gene expression. In a next step, we will shift the purpose of the model from interpretation to prediction. That is, we will use this model to make different predications in different conditions and then help biologists to have an idea of detailed mechanisms of complicated biological systems, and to design experiments to validate the predictions obtained by our simulation.
